# Investigation of Mechanisms in Bone Conduction Hyperacusis With Third Window Pathologies Based on Model Predictions

**DOI:** 10.3389/fneur.2020.00966

**Published:** 2020-09-02

**Authors:** Stefan Stenfelt

**Affiliations:** Department of Biomedical and Clinical Sciences, Linköping University, Linköping, Sweden

**Keywords:** third window, bone conduction, semi-circular canal dehiscence, model, air-bone gap

## Abstract

A lumped element impedance model of the inner ear with sources based on wave propagation in the skull bone was used to investigate the mechanisms of hearing sensitivity changes with semi-circular canal dehiscence (SSCD) and alterations of the size of the vestibular aqueduct. The model was able to replicate clinical and experimental findings reported in the literature. For air conduction, the reduction in cochlear impedance due to a SSCD reduces the intra-cochlear pressure at low frequencies resulting in a reduced hearing sensation. For bone conduction, the reduced impedance in the vestibular side due to the SSCD facilitates volume velocity caused by inner ear fluid inertia, and this effect dominates BC hearing with a third window opening on the vestibular side. The SSCD effect is generally greater for BC than for AC. Moreover, the effect increases with increased area of the dehiscence, but areas more than the cross section area of the semi-circular canal itself leads to small alterations. The model-predicted air-bone gap for a SSCD of 1 mm^2^ is 30 dB at 100 Hz that decreases with frequency and become non-existent at frequencies above 1 kHz. According to the model, this air-bone gap is similar to the air-bone gap of an early stage otosclerosis. The normal variation of the size of the vestibular aqueduct do not affect air conduction hearing, but can vary bone conduction sensitivity by up to 15 dB at low frequencies. Reinforcement of the OW to mitigate hyperacusis with SSCD is inefficient while a RW reinforcement can reset the bone conduction sensitivity to near normal.

## Introduction

In normal function of hearing, the ear canal sound pressure is transmitted to the inner ear via the tympanic membrane (TM) and middle ear ossicles. This results in a motion of the stapes in the oval window (OW) that is mimicked in terms of fluid displaced by the motion of the round window (RW), but with opposite phase (1). The equality of fluid displacement at the two windows indicates that the inner ear space is constant and no other in- or outlet displaces fluid. However, this does not mean that there are no other possibilities for fluid displacement in the inner ear beside the OW and the RW. There are two narrow ducts, the cochlear aqueduct close to the RW and the vestibular aqueduct in the vestibule that connect the inner ear with the fluid in the cranial cavity. Also, blood vessels and neural tissue entering the inner ear may transmit pressure in and out of the inner ear. All these small channels were collectively referred to as the third window by Ranke et al. (2). But for air-conduction (AC) hearing in normal ears, the impedance of these narrow channels are much greater than the impedances of the inner ear fluids, basilar membrane (BM), OW, and RW (3, 4), and they do not affect the volume velocity exciting the BM.

When the stimulation is by bone conduction (BC) (5), i.e., as a vibration to the skull, the equality between the fluid flow at the OW and RW no longer hold (1). One reason for this is that during BC, the bone encapsulating the inner ear moves resulting in a volume alteration of the inner ear space. Another reason is the ability for volume velocity to flow through the vestibular aqueduct at low frequencies (4). Consequently, the vestibular aqueduct facilitates BC hearing at low frequencies when the OW is immobile, for example in ears with otosclerosis (4).

Abnormal conditions exists where a pathological third window arise. The most common such pathological third window is in dehiscence of the semi-circular canal (SSCD) or an enlarged vestibular aqueduct, known as large vestibular aqueduct syndrome (6). Common for these pathologies are that the third window component appears at the vestibular side of the BM, which is important for the hearing outcomes. Symptoms of a third window are decreased sensitivity to low-frequency external sounds while increasing sensitivity to low-frequency internal sounds. This means that AC sound thresholds are elevated at low frequencies while the BC thresholds improve at low frequencies (BC hyperacusis) resulting in a low-frequency air-bone gap (ABG) (7). Other manifestations of a third window in SSCD is autophony (hearing one's own voice as loud or distorted) as well as pulsatile tinnitus and hearing of one's own footsteps (8). Even hearing of eye movements has been reported (9). However, the most severe problem is sound induced vertigo (7), but in the current study only the effects on hearing will be studied.

The low-frequency effect on the AC hearing has been well-investigated in clinical studies (8, 10–12), animal experimental studies (13), cadaveric temporal bone studies (14, 15), and mathematical modeling (16). A usual explanation of the low-frequency AC threshold worsening in SSCD is that the open communication between the vestibule and the cranial space through the semi-circular canal allow sound energy to leak out through this open pathway instead of going to the RW and thereby stimulating the BM. Even if this may serve as a conceptual explanation, it is physically incorrect. The reason for the reduced low-frequency stimulation is that the opening on the vestibule side reduces the cochlear impedance at the OW which leads to a reduction of the intra-cochlear sound pressure that drives the vibration of the BM. This has been shown in intra-cochlear pressure measurement studies on SSCD in cadaveric temporal bones (14, 17).

To mitigate the effects of SSCD in severe cases, surgery can be performed with the aim of sealing the third window, often by plugging the semi-circular canal (18). This is an invasive surgery and others have suggested to reinforce the RW and sometimes also the OW to reduce the effect of the pathological third window (19–21). So far, the outcomes from such reinforcements are unclear and the mechanisms behind the intervention have not been investigated in detail.

The low-frequency effects on BC hearing in SSCD are not equally well-understood as the AC effects. The manifestation of increased low-frequency BC sensitivity, termed BC hyperacusis, is well-established (7, 8) but the mechanisms for this improvement has not been investigated in detail. One suggestion is that the reduced impedance in the vestibule side of the inner ear enhances the volume velocity in the inner ear during BC, known as BC fluid inertia (16, 22). But other mechanisms have also been suggested such as sound pressure transmission from the cranial cavity (8) or that the reduction of the impedance at the OW leads to greater impedance difference between the two sides facilitating BC excitation of the inner ear. Kim et al. (16) investigated the BC inertial effects from SSCD in a finite element model of the ear and found an increased low-frequency BC response. Stenfelt (4) used a lumped-element model to simulate the BC effects from SSCD and reported a low-frequency enhancement. The limitation of both these studies was that they only included one or two contributors to the BC excitation and thereby excluded several other possible contributors. In a later study, the inner ear model by Stenfelt (4) was expanded to include five contributors of BC that have been suggested to be the most important (5, 23).

The aim of this study is to adapt the model in Stenfelt (24) to simulate the effects of inner ear third windows on the five contributors for BC hearing and also for AC hearing. More specifically, the third window effects being studied is dehiscence of the superior semi-circular canal and size variations of the vestibular aqueduct. In addition, the effect from reinforcement of the RW and OW on the predicted hearing results in SSCD is investigated.

## Materials and Methods

### The Model

**Figure 1:**
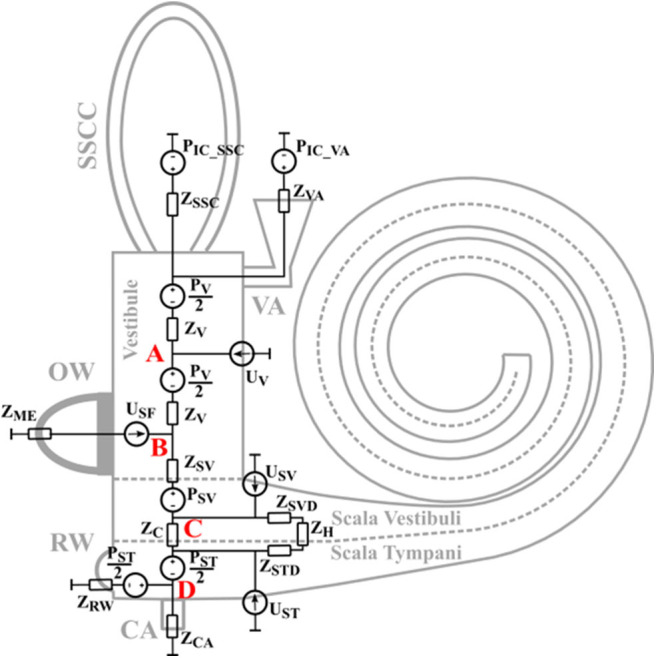
The layout of the lumped element impedance model. The different impedances and sources relate to the physical parts of the inner ear at their approximate positions.

The basis for the model is that the BC vibration travels as a longitudinal one-dimensional wave in the skull bone. Due to the speed of the propagating wave, different positions vibrate with different phases. As the size of the model is ~9 mm, attenuation of the bony wave is neglected. This means that all bony parts move with the same amplitude and direction, but differ in phase. This leads to inertial effects and compressional effects. The inertial effects are caused by the mass and acceleration, and are modeled as pressure sources. The compression effects are caused by space alterations due to phase differences of the vibration while the fluid is considered incompressible, so the volume of the space change leads to a net flow of that volume (4). This is modeled as a volume velocity source. The entire layout of the model is depicted in Figure 1 including the impedances and sources. The skull bone vibrations are taken from the Stenfelt and Goode (25) study as in the previous models.

The sources *P*_*V*_, *P*_*SV*_, and *P*_*ST*_ in Figure 1 are the sound pressures from the fluid inertia in the vestibule, scala vestibuli, and scala tympani, respectively. The *P*_*IC*_*VA*_ is the sound pressure in the CSF at the vestibular aqueduct opening and the P_*IC*_*SSC*_ is the sound pressure in the CSF at the SSCD. These two sources are the intracranial pressure in the CSF and are modeled equal due to physical closeness. There is no pressure source at the cochlear aqueduct since its contribution was found insignificant compared to the contribution from *P*_*IC*_*VA*_ in Stenfelt (24).

The *U*_*SF*_ is a volume velocity source that represents the volume velocity from the stapes motion in the OW when simulating middle ear inertia effects and sound pressure in the ear canal. During AC stimulation, *U*_*SF*_ depends on the sound pressure at the TM, a modeled middle-ear transfer function relating stapes velocity to the sound pressure at the TM (26) when the middle-ear is loaded by the inner-ear model of Figure 1, and an averaged stapes footplate area of 3.85 mm^2^ (27). The same computation is done for the BC external ear component where the ear canal sound pressure in an open ear with BC stimulation is taken from Stenfelt et al. (28). The use of data from occluded ear canals would increase the external-ear contribution to the predicted hearing results. *U*_*SF*_ is based on the finite element modeling in Homma et al. (29) when simulating middle ear inertia. This is different from the previous models where the stapes vibration in Stenfelt et al. (30) was used. The benefit of using the data in Homma et al. (29) is that in the measurements of Stenfelt et al. (30), the motion of the stapes may have been influenced by a combination of different BC mechanisms, while the model motion computed by Homma et al. (29), was only driven by middle-ear inertia.

**Figure 2:**
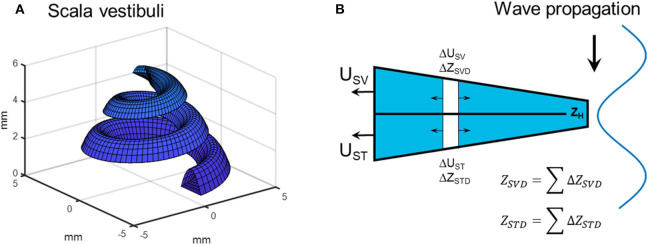
**(A)** Scala vestibuli as represented by the parametrized geometry. **(B)** The computation of the compression fluid flow based on estimations of small section where the contribution to *U*_*SV*_ and *U*_*ST*_ from Δ*U*_*ST*_ and Δ*U*_*SV*_ is based on the wave motion of the bone and the impedances of the ducts as well as on the cochlear load.

The most significant change from the previous models is the computations of the volume velocity sources *U*_*SV*_ and *U*_*ST*_. In the previous models the compression of scala vestibuli and scala tympani was computed in a straight tapered cochlea. In the current model, the estimation of the compressional volume velocity is computed in parameterized coil-shaped ducts (Figure 2A). The cross sectional areas of scala tympani and scala vestibuli are modeled as half elliptic where the scala vestibuli width being 1.8 mm and height being 1.2 mm at the base. These dimensions are linearly reduced toward the apex where the width is 1.6 mm and the height is 0.6 mm. The width and height are for scala tympani 2.5 and 1.4 mm, respectively, that become 1.6 and 0.6 mm at the apex. The radius of the outer part of the coiled cochlea is 5 mm at the base that is reduced to 1.6 mm at the apex. The compression is then computed for consecutive 5 degree-wide sections of the coiled cochlea, leading to 180 sections over the 2.5 turns of the cochlea. For each section, based on the space alteration due to the wave propagation, the volume velocities (Δ*U*_*SV*_ and Δ*U*_*ST*_) of each section together with the impedances (Δ*Z*_*SVD*_ and ΔZ_*STD*_, mass of a tube) are computed (Figure 2B). Based on the impedances of the scala vestibuli and scala tympani ducts (*Z*_*SVD*_ and *Z*_*STD*_, Figure 2B), and the cochlear impedances in Figure 1, all sections' contributions to *U*_*SV*_ and *U*_*ST*_ are computed and summed resulting in a final contribution of the volume velocity from *U*_*SV*_ and *U*_*ST*_ (Figure 2B). The impedance of the helicotrema (*Z*_*H*_, Figures 1, 2) is taken from Marquardt and Hensel (31). The volume velocity source of the vestibule (*U*_*V*_) is computed similar as in the previous model based on the length of the vestibule (5.8 mm) and an elliptic cross-sectional surface area (radius 1.55 and 2.45 mm).

The impedances in Figure 1 are either based on the geometry and material properties, or taken from the literature. The impedance of the middle ear seen from inside the OW, *Z*_*ME*_, is obtained from Puria (32) as well as the impedance of the BM. Also included in *Z*_*C*_ is the fluid mass on both sides of the BM, here modeled as *M*_*SV*_/2 and *M*_*ST*_/2. The other part of the fluid mass in scala tympani is included in the *Z*_*RW*_ impedance that also comprises the stiffness of the RW membrane, a value obtained from Merchant et al. (33). *Z*_*SV*_ is half the mass of scala vestibuli and *Z*_*V*_ is half the mass of the vestibule.

### Third Window

The third window is collectively modeled by three impedances where *Z*_*CA*_ represents the cochlear aqueduct, *Z*_*VA*_ the vestibular aqueduct, and *Z*_*SSC*_ the superior semicircular canal. The position of *Z*_*CA*_ is between the RW and the BM, and has half of the scala tympani mass on each side. *Z*_*CA*_ is modeled as a straight tube of 10 mm with a diameter of 0.15 mm based on the geometry provided in Gopen et al. (3). The impedance of the vestibular aqueduct changed from the previous models and is here based on the geometries presented in Kämpfe Nordström et al. (34). According to their study, the vestibular aqueduct can be characterized as a two part system where the first part is a 2.3 mm straight tube with 0.3 mm diameter. The second part has a horn-like geometry that is 5.7 mm long extending from the first part, has an elliptic cross-sectional area and an end-opening with radius 3.25 and 0.27 mm. The impedance of the second part was computed by successively adding 0.1 mm sections where the impedance was based on the average cross section area.

The impedance of the SSCD was modeled as a hole in the middle of the semi-circular duct. According to Ifediba et al. (35), the area of the superior semi-circular canal is ~2 mm^2^ close to the common crus and vestibule, and 1 mm^2^ at the middle, with a total length of 12 mm. The impedance of the hole between the SSC and the cranial cavity was modeled as a 1 mm long tube with an elliptic cross-section surface where the length of the ellipse was twice the width. This meant that for a SSC without dehiscence, *Z*_*SSC*_ was modeled as two 6 mm tubes with areas diminishing from 2 to 1 mm^2^ in parallel, terminated by the hole with an infinite impedance (no hole). As the hole became greater, the length of the semi-circular ducts became shorter by the length of the larger radius of the hole. In the current study, the maximum size of the hole had a larger diameter of close to 2 mm, corresponding to a cross-section area of 6 mm^2^, and the semi-circular ducts were then reduced to ~5 mm long. Consequently, the length of the two parallel semi-circular ducts varied between 5 mm for the largest size hole and 6 mm for the no-hole condition. Table 1 list all impedances.

**Table 1:** The values of the impedances in Figure 1.

**Impedance**	**Value**
*Z_*SSC*_*	$j\omega \cdot \left(2.83\cdot 10^{6}+\frac{4\cdot 10^{-6}}{3\cdot A_{D}}\right)+3.9\cdot 10^{7}+\frac{\pi \cdot 8\cdot 10^{-6}}{A_{D}^{2}}$
*Z_*VA*_*	*jω* · 5.68 · 10^7^ + 1.27 · 10^10^
*Z_*V*_*	*jω* · 2.43 · 10^5^
*Z_*ME*_*	$j\omega \cdot 4.4\cdot 10^{5}+1.2\cdot 10^{12}+\frac{8.1\cdot 10^{13}}{j\omega }$
Z_SV_	*jω* · 2.45 · 10^5^
Z_SVD_	*jω* · 2.86 · 10^7^
Z_STD_	*jω* · 2.14 · 10^7^
*Z_*H*_*	$\left(j\omega \cdot 1.7\cdot 10^{7}+2\cdot 10^{8}\right)//\left(2.2\cdot 10^{9}+\frac{1.59\cdot 10^{12}}{j\omega }\right)$
*Z_*C*_*	*jω* · 10.02 · 10^5^ + 10^10^
*Z_*RW*_*	$j\omega \cdot 4.59\cdot 10^{5}+5\cdot 10^{8}+\frac{7\cdot 10^{12}}{j\omega }$

*“//” means parallel computing, A_D_, area of dehiscence in m^2^*.

### Simulations

The simulations were conducted using the principal of superposition, where the model was solved for each particular BC stimulus path by turning on all of the sources associated with each BC stimulus mode or AC stimulation and turning the others off. Once the contribution of each stimulus path to the hearing result has been computed, the amplitude squared of the different contributions are summed to compute a quantity proportional to sound power that is used to define the overall hearing result. A more realistic summation would be to add the amplitude and phase of the individual components; however, the phase response of the complex three-dimensional vibration of the real head is not represented in the current model. The computation of a dB change is done according to equation 1

(1) $$\begin{array}{r} dB=10\cdot \log_{10}\left(\frac{A_{sum}^{2}}{A_{ref}^{2}}\right) \end{array}$$

where $A_{sum}^{2}$ is the sum of the contributors' squared amplitudes after the manipulation and $A_{ref}^{2}$ is the sum of the contributors' squared amplitudes before the manipulation.

It is assumed that the drive of the BM, and thereby the hearing excitation, is caused by the volume velocity through *Z*_*C*_ in the model. This is proportional to the sound pressure difference between scala vestibuli and scala tympani, which has previously been argued to be the drive of the cochlea (14, 17). Therefore, the flow through *Z*_*C*_ is used to compare the contributions from each component and also to investigate changes between conditions.

First, the model itself is validated against experimental data in the normal condition. This is accomplished by comparing intra-cochlear pressures in the model with measurements in cadaveric temporal bones with AC stimulation (14, 36) and BC stimulation (37) and also with BC stimulation in whole human cadaver heads (38). In the experimental datasets the scala vestibuli and scala tympani pressures were measured by small pressure probes inserted into the scalae through tiny holes that were sealed during the measurements (14, 36–38).

The intra-cochlear pressures depend on the exact position of the probes inside the inner ear. In the model, intra-cochlear pressure were extracted at four positions defined as A to D in Figure 1. Position A is in the center of the vestibule, position B is at the border between the vestibule and scala vestibuli close to the stapes footplate, position C is at the center of scala vestibuli, and position D is at the center of scala tympani. According to the descriptions of the experiments in the temporal bones, the probe positions were close to positions B and D in the model (14, 36, 37). The exact position of the pressure sensors were not equally well-defined in the Mattingly et al. (38) study. In the AC stimulation comparisons, the ear canal sound pressure is used as reference while the cochlear promontory velocity is used as reference for the BC stimulation compairsons.

Beside the normal condition of the ear, three different conditions are investigated. The first condition is the effect on the AC contribution and the five BC contributors when a hole appears in the superior semi-circular canal, where the hole dimension goes from no-hole to a hole size of 6 mm^2^. The second condition explored is a change of the size of the vestibular aqueduct. A large variability is noted in the anatomical studies of the vestibular aqueduct (34), and its effect on the cochlear excitation is examined for variations between half the diameter of the small duct to twice the diameter of the small duct. The third condition investigated in the current study is the effect of OW and RW reinforcement with a present SSCD. This is modeled by increasing the stiffness of the OW and RW separately and jointly, where the increase of the stiffness was either 10 times or 100 times.

## Results

### Model Validation

**Figure 3:**
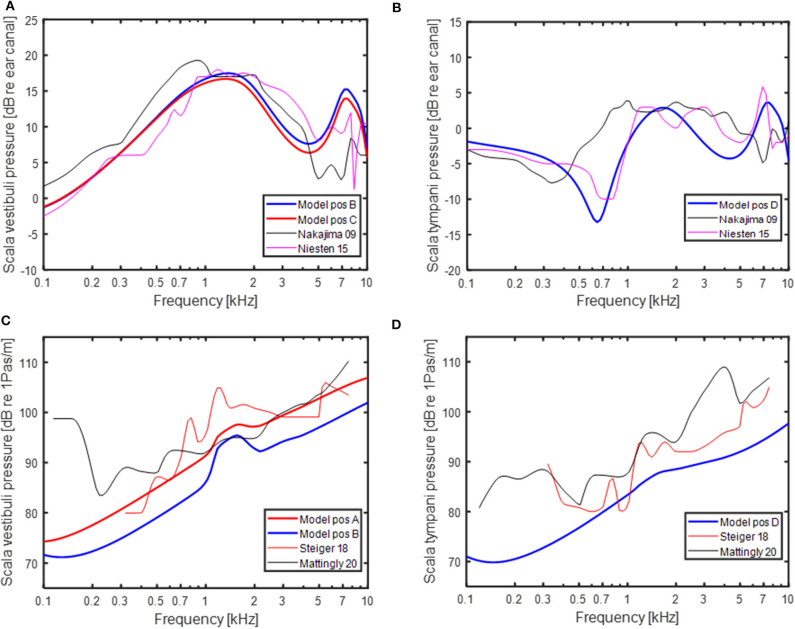
Predictions of intra-cochlear sound pressures with AC stimulation and experimentally measured intra-cochlear sound pressures in Nakajima et al. (36) and Niesten et al. (14) in **(A)** the vestibular side and **(B)** scala tympani. Predictions of intra-cochlear sound pressures with BC stimulation and experimentally measured intra-cochlear sound pressures in Stieger et al. (37) and Mattingly et al. (38) in **(C)** the vestibular side and **(D)** scala tympani. Positions **(A–D)** refer to position in the model schematics in Figure 1.

The model predictions were compared to experimentally obtained intra-cochlear sound pressures. With AC stimulation, the sound pressures in scala vestibuli and scala tympani in relation to a sound pressure in front of the ear drum are shown in Figures 3A,B, respectively. The model-predictions of AC driven intra-cochlear sound pressures at position A and B were nearly identical, and only the sound pressures at positions B and C are included in Figure 3A. The sound pressure differences between position B and C are small with almost no difference at the lower frequencies and ~2 dB lower pressure levels for position C compared to position B at frequencies above 1 kHz. The sound pressures at both positions are in general agreement with the experimentally obtained scala vestibuli sound pressures shown in Figure 3A, where the difference between the model-predicted sound pressures and experimentally obtained sound pressures are similar to the difference between the two experimentally obtained sound pressures. The model-predicted scala tympani sound pressure in Figure 3B is in line with the experimentally obtained sound pressures. The model predictions in Figure 3B are most similar to the Niesten et al. (14) data while the model predicts 5 to 10 dB lower sound pressure at frequencies between 0.5 and 1.0 kHz compared with Nakajima et al. (36).

The model-predicted sound pressures with BC excitation are shown in Figures 3C,D together with experimentally obtained BC stimulated intra-cochlear sound pressures in Stieger et al. (37) and Mattingly et al. (38). The scala vestibuli side intra-cochlear sound pressures in relation to the cochlear promontory velocity are shown in Figure 3C. The sound pressures at position B and C were within a couple of dBs and only the sound pressures at positions A and B are provided. The sound pressure at position A is relatively close to the experimentally obtained sound pressures while the sound pressure at position B is around 5 dB lower than the position A sound pressure. The model based sound pressure predictions and the Mattingly et al. (38) experimental data indicate an overall 20 dB/decade rise while the Stieger et al. (37) data show a steeper rise at frequencies below 1.5 kHz and a near flat response with frequency at the higher frequencies. The BC model predictions of the scala tympani sound pressure in Figure 3D is relatively similar to the Stieger et al. (37) measurements while the Mattingly et al. (38) sound pressures are 5 to 10 dB greater compared to the model predictions.

### Model Prediction of BC Contributors

**Figure 4:**
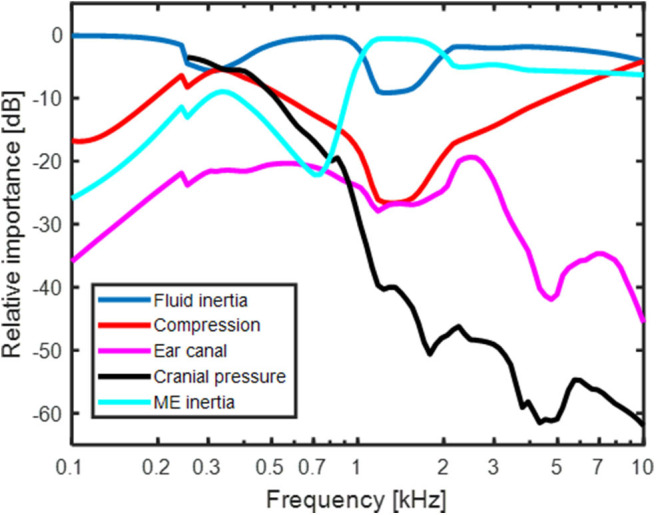
The relative importance from the five contributors of BC sound in a healthy ear.

The result in Figure 4 shows the predicted relative contribution from the five components for BC excitation of the BM in a healthy ear. The general trends are similar to the predictions in Stenfelt (24) with the exception for a few details. The overall most important contributor in the healthy ear is fluid inertia (blue line in Figure 4). The middle ear inertia has its major contribution at frequencies between 1 and 2 kHz which is also the frequency range where the middle ear ossicles has its resonance with BC stimulation (29, 30). The use of Homma et al. (29) modeling data for the current simulations increased the middle ear inertia importance around its resonance compared to the earlier model where the Stenfelt et al. (30) data were used. Another difference seen between the current and previous models is the predicted contribution from cochlear compression (red line in Figure 4). The use of a coiled cochlea reduced the contribution at the lowest (below 300 Hz) and mid-frequencies, while increasing its contribution at the highest frequencies. The relative contribution from the ear canal sound pressure and intra-cranial pressure is similar to the previous study. It should be noted that the intracranial pressure used here from the Roosli et al. (39) is not the sound pressure in the CSF close to the inner ear but obtained intra-cranially in cadaver heads where the brain was replaced by fluid. Due to uncertainties in the measurements, only CSF pressures at frequencies above 250 Hz is used. However, according to the trajectory, the pressure transmission from the CSF may be important at low frequencies.

### Model Predictions of SSCD

**Figure 5:**
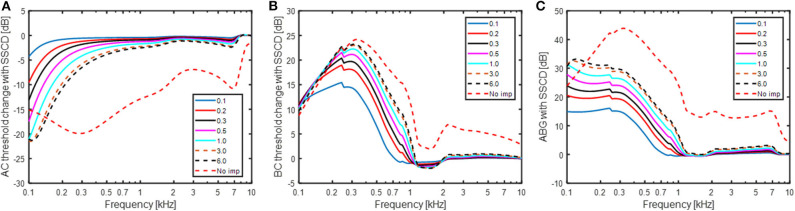
Estimations of **(A)** AC threshold change, **(B)** BC threshold change, and **(C)** ABG for a SSCD based on the model in Figure 1. A negative value means decreased sensitivity while a positive value means improved sensitivity in panels **(A,B)**. The legend indicates the dehiscence area in mm^2^ and “No imp” refers to the condition where *Z*_*SSC*_ = 0.

Figure 5 shows the simulated effect of a SSCD with different sizes of the dehiscence. The dehiscence size goes from 0.1 to 6 mm^2^ and an additional simulation termed “*No imp*” is included that represent the case when *Z*_*SSC*_ is zero. This can be seen as a theoretical bound on the greatest change achievable by a SSCD, for example a large hole close to the vestibule. In Figure 5A, the predicted effect on the AC threshold is shown in relation to a healthy ear. It should be noted that a negative value means worse hearing and a hearing threshold would be increased by that dB level. The AC predictions show a gradually increase in the low-frequency reduction with increasing dehiscence area, but the effect of increased area is small for areas > 3 mm^2^. The predicted AC threshold changes are primarily seen at frequencies below 500 Hz where a hole of 1 mm^2^ gives a reduction of 3 dB while it results in a reduction of 16 dB at 125 Hz. Figure 5B shows the simulated improvement in BC hearing from the SSCD. It indicates a relatively abrupt increase of ~15 dB at 250 Hz for the smallest dehiscence simulated, and the increase rises with dehiscence dimension up to 23 dB at 300 Hz, the frequency with maximum predicted threshold change. It is noteworthy that the simulations predict less increase at the lowest frequencies and at 100 Hz the increase is ~10 dB independent of dehiscence dimension.

The predicted ABG is indicated in Figure 5C which is the difference between Figures 5A,B. The overall morphology shows an increase of the predicted ABG with decreasing frequency between 300 and 1,000 Hz, while the ABG is nearly constant at frequencies below 300 Hz. The predicted ABG has a maximum of 15 dB for the smallest dehiscence simulated (0.1 mm^2^) and increases with increasing dimension of the dehiscence reaching just over 30 dB for the largest dehiscence (6 mm^2^). In the “No-imp” condition, the maximum ABG reaches 45 dB at 300 Hz.

**Figure 6:**
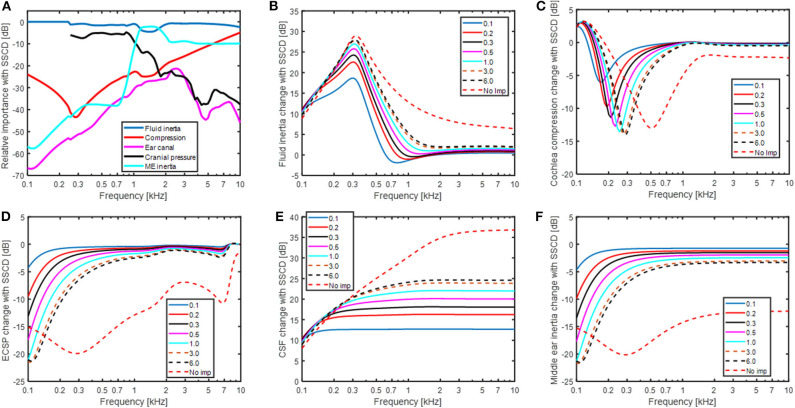
**(A)** The relative importance for the five contributors to BC sound in a SSCD ear with a dehiscence area of 6 mm^2^. **(B–F)** The changes of each individual contributor to BC sound for different areas of a SSCD given in mm^2^.

The predicted changes in the BC contributors with SSCD are shown in Figure 6. Figure 6A show the relative contribution of the five BC components when the SSCD is 6 mm^2^. Compared to Figure 4 that shows the relative contributions for BC in the healthy ear, the fluid inertia has become even more dominant. The differences seen are, as expected, at frequencies below 1 kHz. The alterations in Figure 6A is a combination of the effects seen in Figures 6B–F. Figure 6B shows the effect for fluid inertia which follows the change in predicted BC thresholds (Figure 5B) closely. This prediction is again consistent with fluid inertia as the most important contributor for BC hearing, also in a pathological ear. One interesting observation is the finding at the lowest frequency (100 Hz) where all simulations of a SSCD starts at 10 dB independent of dehiscence size and increases with frequency up to 300 Hz. This is a result of the RW stiffness that restricts the motion of the fluid over the BM at these low frequencies. Consequently, according to this model, the maximum improvement from fluid inertia at frequencies below 300 Hz is determined by the RW stiffness. The inner ear compression component (Figure 6C) show nearly an opposite function compared to the fluid inertia, but with less impact. A SSCD result in a decreased BM stimulation from the compression with a minimum of between−5 and−15 dB at frequencies between 200 and 300 Hz. The reduction is primarily a result of *U*_*V*_ and *U*_*SV*_ directing the volume velocity toward the SSC instead of over the BM.

The effect of sound pressure in the ear canal with BC (Figure 6D) and middle ear inertia (Figure 6F) show nearly identical results as with AC stimulation in Figure 5A since they all stimulate the inner ear via the stapes velocity (*U*_*SF*_) in the model. The low-frequency reduction coincides with the decrease in cochlear impedance seen at the OW (omitting *Z*_*ME*_). Since the stapes velocity alteration with the change in impedance is small, the reduced cochlear impedance leads to a reduced intra-cochlear sound pressure that decreases the BM excitation. The effect of sound pressure transmission from the CSF to BM vibration (Figure 6E) shows the greatest deviation from the other contributors having the greatest impact at the higher frequencies. The increase at 100 Hz is ~10 dB for all SSCDs simulated that increase further at higher frequencies and a larger dehiscence result in a greater change with around 13 dB for the 0.1 mm^2^ dehiscence, 25 dB with 6 mm^2^ dehiscence, and 37 dB in the “*No imp”* condition.

### Model Predictions of Vestibular Aqueduct Variations

**Figure 7:**
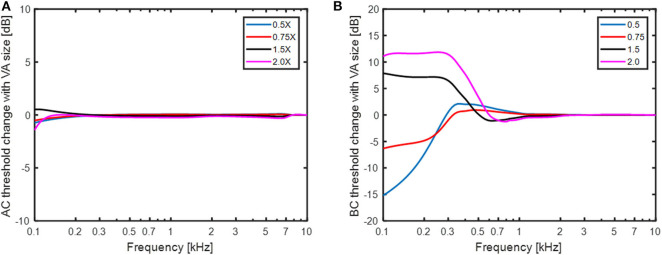
The change in **(A)** AC thresholds and **(B)** BC thresholds when the size of the small duct in the vestibular aqueduct is altered between half diameter (0.5 X) and double diameter (2X).

The predicted AC and BC threshold changes with varying size of the vestibular aqueduct are shown in Figure 7. In the simulations, the narrow part of the vestibular aqueduct that was modeled as 2.3 mm long tube with a diameter of 0.3 mm was altered with diameters between 0.15 mm (half) and 0.6 mm (double). Since this narrow duct dominates the impedance of the vestibular aqueduct, only the initial cross sectional area of the second horn-like part was altered since it had the same area as the narrow tube. Figure 7A indicates that this range of variation did not affect the simulated AC thresholds. The greatest predicted change was around 1 dB appearing at 100 Hz. The predicted BC thresholds in Figure 7B were more affected by the dimension of the vestibular aqueduct. In these simulations, the impedance of the semi-circular canal (*Z*_*SCC*_) was infinite and the only volume velocity possible between the vestibule and the cranial cavity was through the vestibular aqueduct. The BC estimations varied between−15 dB and 12 dB at the lowest frequencies, primarily below 500 Hz. A smaller vestibular aqueduct size reduced the predicted BC sensitivity while a greater vestibular aqueduct size improved the predicted BC sensitivity. The explanation for the change in BC sensitivity with vestibular aqueduct alteration is the same as with SSCD, a reduced impedance allow more fluid to be displaced by the fluid inertia thereby improving the BC excitation, while an increase in the impedance restricts the fluid inertia. The middle ear inertia and sound pressure from the ear canal is affected similar as the simulated AC thresholds (Figure 7A) while the cochlear compression shows a small low-frequency decrease with larger vestibular aqueduct and a small increase with smaller ducts. The CSF pressure transmission decreases by 12 dB with halving the duct diameter and improves by 10 dB with doubling the duct diameter, almost independent of frequency. These results indicate that the vestibular aqueduct is important for low frequency BC hearing.

### Model Predictions of Cochlear Window Reinforcement

**Figure 8:**
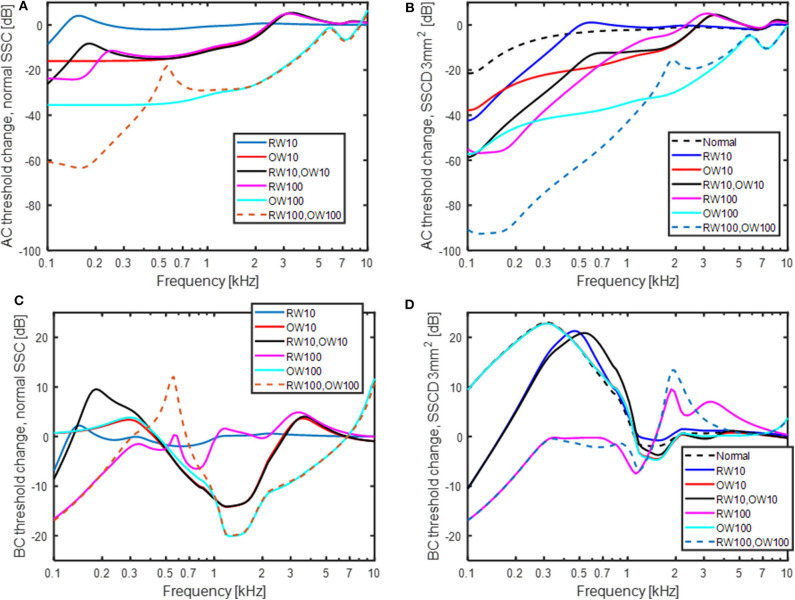
The effect of increasing the stiffness of the OW and RW 10 and 100 times in isolation and jointly. **(A)** AC thresholds with normal semi-circular canal, **(B)** AC thresholds with a SSCD of 3 mm^2^, **(C)** BC thresholds with normal semi-circular canal, and **(D)** BC thresholds with a SSCD of 3 mm^2^. “Normal” in the legend means no change of OW and RW stiffness.

The predicted effect of reinforcement of the OW and RW, modeled as an increase in the stiffness, is shown in Figure 8. The predicted effect on the AC thresholds is shown in Figure 8A for a healthy (no SSCD) ear when the RW and OW stiffness is increased by 10 or 100 times, in isolation or jointly. A stiffness increase of the OW affects the predicted AC thresholds more than a stiffness increase of the RW, where a 10 times increase of the OW stiffness result in similar AC threshold depression as a 100 times increase in the RW stiffness. The greatest decrease is when both the RW and OW stiffnesses are increased 100 times, resulting in around 60 dB worse predicted thresholds at the lowest frequencies. The predicted result shown in Figure 8B is the change in AC thresholds compared with a normal healthy ear, when a SSCD of 3 mm^2^ coincides with the alteration of OW and RW stiffness. The normal curve in Figure 8B (black dashed line) is the result without changing the stiffness of the RW or OW but with a SSCD of 3 mm^2^, i.e., the same as the 3 mm^2^ curve in Figure 5A. The SSCD boosts the effect from stiffening the RW and OW and a 10 times stiffening of the RW causes a significant predicted AC hearing reduction at the low frequencies. Now, the OW stiffening result in greater reduction at the mid frequencies but affects the lowest frequencies similar as a stiffening of the RW. The combined effect of increasing both the OW and the RW stiffness 100 times result in a reduction of simulated AC hearing by more than 90 dB at the lowest frequencies.

The predicted effects on BC thresholds with OW and RW stiffness increases are shown in Figures 8C,D. In the healthy ear (Figure 8C), stiffening the RW 10 times gives nearly no effect and stiffening the RW 100 times gives a threshold depression at frequencies below 300 Hz amounting to 17 dB at 100 Hz. Increasing the stiffness of the OW has no effect at the lowest frequencies but decreases the predicted BC thresholds at mid frequencies. Increasing the OW stiffness reduces the inertial contributors and the result is primarily from the cochlear compression component. The effect with OW and RW stiffness increase is different with SSCD shown in Figure 8D. In this case, increasing the OW stiffness has no impact and the curves for 10 times and 100 times stiffness increase falls on top of each other and are nearly identical with the “no stiffness change” curve (here termed normal). Increasing the RW stiffness affect the predicted BC thresholds by reducing the low-frequency BC sensitivity. When the RW stiffness is increased 100 times, the simulated BC thresholds are close to normal at frequencies above 250 Hz and a reduction of 17 dB at 100 Hz is seen.

## Discussion

### The Model

The current study has investigated the impact from pathological third windows on AC and BC thresholds, and also investigated the mechanisms for the changes. Both the strengths and the weakness of this study are that it is based on a computational model. The strength is that a model facilitates investigations of the mechanisms underlying the results. The weakness of the model is that it is just a model, a simplification of the reality. All models have their limitations and so does this model. For example, the AC transmission is limited to ossicular vibration only and no effect of sound pressure in the middle ear cavity is included. This means that other AC pathways that may become important when the ossicular chain transmission is restricted is omitted in the current model. The model is similar to other lumped-element models of the inner ear where the stimulation is by AC (31, 40, 41). Moreover, the model could predict intra-cochlear sound pressures obtained experimentally in cadaveric temporal bones when the stimulation was a sound pressure in the ear canal (Figures 3A,B).

The greatest uncertainty is probably in the representation of the BC model. Models that simulate general BC responses are rare. Most are restricted to a single mode of excitation and have a simplified geometry (42, 43). Whole-head models for BC have been developed (44, 45) but do not include the detailed structures of the inner ear, for example the vestibular and cochlear aqueducts, and is therefore inappropriate for the current study. Even if there are uncertainties with the current model for BC excitation, it has been revised from previous versions (4, 24) by updated geometries, impedances, and excitation patterns, and continue to show similar results.

The model's ability to predict experimental and clinical findings with BC stimulation has been shown in a previous study (4). In the current study, the model validation was done by comparison to experimentally obtained intra-cochlear pressures (Figures 3C,D). The model predictions of the intra-cochlear pressures showed similar frequency responses as the experimentally obtained intra-cochlear pressures, but some 5–10 dB overall lower levels. One difference between the model simulations and the experimental measurements with BC stimulation is that the model is restricted to a one-dimensional vibration behavior while the experimental data are obtained with vibration in all three dimension, even if the cochlear promontory vibration is reported as a one-dimensional velocity. The bone encapsulating the inner ear vibrates in all three space dimensions with nearly identical magnitudes (25). Consequently, in the experimental measurements the contribution from three orthogonal vibration directions are summed in the cochlea increasing the overall pressure compared to a one-dimension stimulation. The summation of these contributors in magnitude and phase is unknown, but the addition in sound power from three orthogonal vibrations of equal magnitude is nearly 5 dB. So, part of the discrepancy between the model predictions of intra-cochlear sound pressures and the experimentally measured intra-cochlear sound pressures is caused by the one-dimensional excitation in the model and three-dimensional excitation in the experiments.

There are additional differences between the model simulation of BC sound and the experimental measurements. In Stieger et al. (37) the measurements are conducted in isolated temporal bones excluding the influence from the external ear and CSF pressure. Even if those contributors are not dominating the BC response according to the model simulations in Figure 4, the extraction of the temporal bone may affect some of the loading impedances, for example the loading from the vestibular aqueduct or the middle ear ossicles. In Mattingly et al. (38), the measurements were conducted in intact cadaver heads, but the pressure sensors were not rigidly attached to the bone by cement but only with alginate. According to Stieger et al. (37), such attachment introduce artifacts in the measurement of intra-cochlear sound pressure with BC stimulation. This fact introduce uncertainty in the compairson to the Mattingly et al. (38) data.

In the computation of the BC response in the model, the five BC pathways' contributions were added in sound power and not with the individual components' amplitude and phase. This can be seen as a weakness of the model and simulations. However, as stated previously, the bone encapsulating the inner ear vibrates in three dimensions (25), and the amplitude and phase relations between these directions is not well-established. Moreover, the amplitude and phase relation between the directions depend on the exact position of the stimulation. Consequently, if the different contributors in the model were to be added with phase, the phase can be very different in reality due to the influence from vibrations in other directions. It was therefore decided to add the sound power from contributors and neglect the possibility that some of the components may add destructively at specific frequencies. Another aspect is that adding the components with amplitude and phase only influences the results when they are of similar magnitude. When investigating the contribution from the five pathways in Figure 4 it can be seen that the BC response is mostly dominated by one component. In such case, including the phase in the addition has a minor influence on the final result.

The greatest difference between the current BC model and the previous version was the coiled cochlea and the compressional volume velocity based on volume changes in small sections of the coiled scalae. The cochlear shape was parameterized to facilitate the estimation of volume change based on phase differences in a more correct anatomy. This novel way of estimating the compression during BC changed the effect of compression response in the healthy ear (Figure 4). Compared to previous model predictions, the low and mid-frequency responses were slightly lower and the high-frequency response was increased. The reduction at low frequencies was mainly due to the ability for the volume velocity to flow through the helicotrema instead of forcing all volume velocity toward the cochlea while at high frequencies, the geometrical distribution increased the volume velocity output.

The computations of the coiled scalae also gave impedances for the cochlear duct (*Z*_*SVD*_, *Z*_*H*_ and *Z*_*STD*_, Figures 1, 2) that were included in the computations for all contributors. This series of impedances have a greater magnitude than the impedance over the BM (*Z*_*C*_, Figure 1) that it parallels. It did not impact the computations at the frequencies investigated here, but may influence results at lower frequencies (15, 31). The other larger alteration was the geometry of the vestibular aqueduct that previously consisted of two serial connected tubes with different length and diameters. It was now made by one straight narrow tube and one horn-shaped part that had an increasing elliptic cross-sectional area (34). However, this change did not significantly alter the responses of the BC predictions in Figure 4. The thinner tube dominates the impedance of the vestibular aqueduct, and it was similar for the two models. The result in Figure 4 indicates that for the healthy ear, fluid inertia and middle ear inertia contributes the most.

The results in this study are based on model simulations and need to be interpreted accordingly. The parameters of the model is based on averages from anatomical and physiological measurements. Hence, an individual can deviate from these average data and show different results. However, the trends should be similar and the mechanisms behind the results should also be the same.

### Hearing Changes With SSCD

Figure 5 show the predicted changes in AC thresholds, BC thresholds, and ABG with a SSCD. The simulations were done for a dehiscence area of up to 6 mm^2^. The limitation to 6 mm^2^ was based on the study by Hunter et al. (46) that reported most dehiscence areas to be 6 mm^2^ or smaller, with mean areas in different studies ranging from 1.44 to 3.19 mm^2^. The ABG in Figure 5C show a monotonic increase with increasing size of the dehiscence at frequencies below 1 kHz. This is in line with reports from clinical and experimental studies (14, 46, 47). Hunter et al. (46) computed the correlation between dehiscence size and ABG at 500 Hz and reported it to be *r* = 0.27. When the ABG at 500 Hz in Figure 5C is related to dehiscence area a correlation coefficient of *r* = 0.77 is obtained, a value significant higher than the clinical observed correlation. It has also been suggested that when the dehiscence sizes becomes greater than the cross sectional area of the semi-circular canal, it does not add any effect to the ABG (22). This is partially corroborated in the current study where only small changes of the ABG occur once the area has reached 1 mm^2^, that is the cross-sectional area of the semi-circular canal for the model (35). Since most sizes of the dehiscence reported clinically are greater than the cross-sectional area of the semi-circular canal (averages ranging between 1.44 and 3.19 mm^2^), only weak relations between the dehiscence size and ABG is expected.

The ABG is the difference between the AC and BC thresholds, and according to the model the AC thresholds affect the ABG most at the lowest frequencies while the BC thresholds affect it mostly between 200 and 500 Hz. ABGs for patients with SSCD have been reported up to 50 dB (47), which is greater than the model predicts. If the “No imp” condition is considered, the maximum ABG at 250 and 500 Hz is ~40 dB. One reason for the limitation of the ABG to ~30 dB in the model is that the position of the dehiscence is modeled at the middle of the semi-circular canal. Williamson et al. (48) reported the positions of the dehiscence to be approximately equally distributed at the three areas arcuate eminence, posterior aspect, and posterior aspect, of the semi-circular canal. Consequently, the clinically observed spread in the position of the dehiscence add variability to the ABG. For example, Songer and Rosowski (22) reported the difference between a dehiscence close to the vestibule and 5 mm from the vestibule to be about 10 dB. This indicates that the difference in the ABG in Figure 5C between 6 mm^2^ and “No imp” of ~10 dB is reasonable and that the model is able to capture ABGs reported clinically.

Niesten et al. (14) reported the intra-cochlear sound pressure in human temporal bones subsequent to SSCD to be reduced 10 to 15 dB at 100 Hz that recovered with frequency and no effect was seen at frequencies above 800 Hz. This is in line with the model predictions in Figure 5A where a worsening of the AC threshold of 10 to 20 dB is predicted at 100 Hz, depending on the dehiscence area, that recovers with frequency and is <3 dB at 800 Hz. In a study on chinchillas, Songer and Rosowski (22) showed an abrupt change in the AC threshold when opening the semi-circular canal. That was not found for the model in the AC threshold, but the BC threshold was altered with up to 15 dB when a hole of 0.1 mm^2^ was introduced in the model. This can be explained by the impedance difference between the SSCD and the vestibular aqueduct (*Z*_*SSC*_ and *Z*_*VA*_) for even a small hole, facilitating fluid inertia at low frequencies. The model predict between 15 and 20 dB BC threshold improvement with a SSCD of 0.5 mm^2^ or larger at frequencies between 125 and 500 Hz. Since most audiometers do not measure BC thresholds better than−10 dB HL, an improvement of 20 dB can be difficult to measure if the patient has no sensorineural hearing deficit. This implies that some clinical studies underestimate the ABG due to insufficient dynamic range for the BC testing. Brantberg et al. (8) circumvented this problem by testing the BC thresholds with a minishaker and a load cell to estimate the vibration force applied. When comparing SSCD patients with normal controls they reported BC threshold improvement with SSCD in the range of 15–23 dB at 125 Hz, 17–20 dB at 250 Hz, 5–19 dB at 500 Hz,−4 to 12 dB at 750 Hz, and−4 to 5 dB at 1,000 Hz. These data are in line with the model predicted BC threshold improvement in Figure 5B. One possible problem is that the data by Brantberg et al. (8) were obtained with occluded ears which may enhance the low-frequency contribution by the ear canal sound pressure which is affected differently by the SSCD than the BC hearing in general (Figure 6) (28, 49).

Since there is a risk of a ceiling effect when clinically measuring BC thresholds in patients with SSCD, and thereby underestimating the ABG, there is a risk of miss-diagnose patients with SSCD as non-pathological. This risk is even greater if the BC thresholds is not obtained at frequencies below 500 Hz. A solution to this problem is to use a BC transducer that can be used at low frequencies, for example the Radioear B81 BC transducer (50), and measure BC thresholds down to 250 Hz but preferable down to 125 Hz. Also, recalibrating the audiometer for BC transducer use so it permits measurement down to−20 dB HL enable a more correct measure of the BC hyperacusis and a more reliable estimation of the ABG and SSCD diagnosis.

### Bone Conduction Contributors With SSCD

The relative importance of the different BC contributors change with the SSCD (cf Figures 4, 6A). In the SSCD ear, the contribution of the fluid inertia dominates the response and only the middle ear inertia contributes at around its resonance frequency. The low-frequency contribution from the middle ear inertia and ear canal sound pressure is reduced similar to the AC thresholds (Figures 6D,F). This is caused by the reduction of the cochlear impedance due to the SSCD. According to Chien et al. (47), the stapes velocity increased by 3–5 dB after the introduction of a SSCD, while the RW motion was reduced by 15 dB at 100 Hz. Consequently, the stapes velocity increase after the SSCD cannot compensate for the low-frequency cochlear impedance decrease and the low-frequency intra-cochlear pressure decreases, which is reflected in the reduced RW motion.

A common complaint by SSCD patients is disturbance by internal sounds such as eye movement, chewing, and bowel sounds (7, 8). It has been hypothesized that internal sounds are transmitted by the intracranial pressure transmission (8). According to the model simulations in Figure 6, the sound pressure transmission from the CSF is not dominating the BC response after SSCD and its frequency function is very different from that observed with BC thresholds. The BC thresholds improves at the low frequencies after a SSCD while the sound pressure transmission from the CSF show the greatest improvement at the highest frequencies (Figure 6E). This indicates that the internal sounds are not transmitted through the SSCD but is a result of the general BC improvement enhancing internal sounds that cause the skull bone to vibrate.

### Hearing as a Function of the Vestibular Aqueduct

The changes in cochlear impedance in the simulations of the vestibular aqueduct in Figure 7 is less dramatic than those with SSCD in Figure 6. This is due to the smaller cross-section area of the vestibular aqueduct compared to the dehiscence areas used to simulate the SSCD. The small tube area of the vestibular aqueduct is 0.07 mm^2^ in the normal condition, 0.28 mm^2^ in the double-diameter condition, and 0.018 mm^2^ in the half-diameter condition. These variations do not affect AC hearing but has an influence on the BC hearing at low frequencies. The standard deviation for the small duct diameter is given in Kämpfe et al. (34) as 0.12 mm which indicates that the normal size +/- 1 SD is almost covered in the 0.5X to 1.5X results. According to the simulations of the BC thresholds, this would indicate a variability of−15 to + 7 dB at 100 Hz. To the author's knowledge, there are no reports on the BC threshold variability at such low frequencies. Clinically, BC thresholds are usually only obtained at 250 Hz and above, and the variation in BC thresholds at 250 Hz due to the spread in vestibular aqueduct size is close to +/- 5 dB, which is lower than the anticipated variability in BC threshold testing (51).

Large vestibular aqueduct syndrome (LVAS) has been reported to result in significant ABGs at frequencies below 1 kHz. Merchant et al. (52) reported LVAS to give an ABG that amounted to 51 dB at 250 Hz that decreased with frequency to 12 dB at 1 kHz. That is far more than what can be expected from the variation of vestibular aqueduct sizes in Figure 7, and is more in line with the “No imp” data in Figure 5C, indicating a larger opening than 2–4 mm^2^ that was modeled as the parallel semi-circular canal. Unfortunately, no data on the size of the LVAS was provided in Merchant et al. (52).

### Round Window and Oval Window Reinforcement

Stiffening the RW and OW affected the predicted AC thresholds more than the BC thresholds (Figure 8). This could be expected as clinically it has been demonstrated that occlusion of the OW [e.g., otosclerosis (53), or RW atresia (54)], affect the AC thresholds significantly but the BC thresholds <20 dB. For AC, increasing the stiffness of the OW had greater effect than the same stiffness increase of the RW. This can be explained by the two stiffness's being in the AC transmission pathway where the OW stiffness is greater than the RW stiffness, and an increase of the dominating stiffness has the greatest influence. Also, it can be noted that the effect of OW and RW stiffness increase has a greater effect in the SSCD condition (Figure 8B) compared to the healthy ear. The predicted loss from the RW stiffness increase in Figure 8A is similar to that estimated in Elliott et al. (41), using a similar type of model.

**Figure 9:**
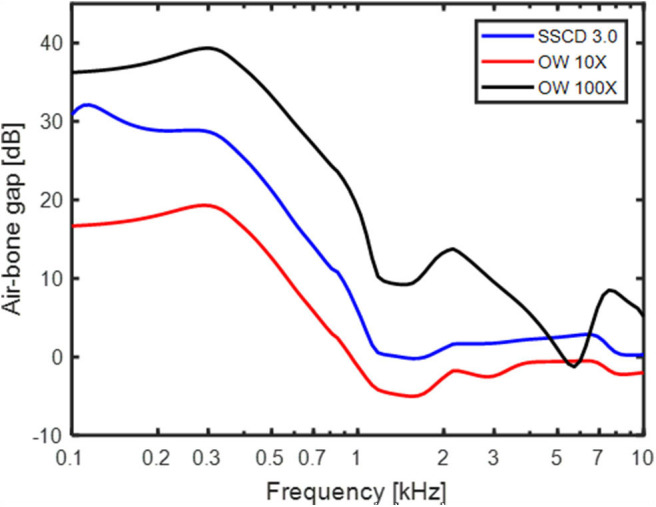
The ABG predicted by the model for a 3 mm^2^ SSCD, OW stiffness increase 10 times, and OW stiffness increase 100 times.

Increasing the stiffness of the RW gives a relatively limited low-frequency effect for the normal ear (Figure 8C) while stiffening the OW gives a mid-frequency lowering of the predicted BC thresholds. The increase in OW stiffness can be seen as a model for otosclerosis where the stapes become immobile. The curve for increasing the OW stiffness 100 times in Figure 8C do mimic the well-known Carhart notch for BC thresholds in an otosclerotic ear (53). This indicates that the Carhart notch is the reduction of the inertial effect so that the cochlear compression dominates the BC response. Moreover, if an increased OW stiffness is seen as a beginning of an otosclerosis, the predicted ABG of early otosclerosis can be obtained from the predicted AC and BC threshold shifts with OW stiffness in Figures 8A,C. Figure 9 shows the predicted ABG for an OW stiffness increase of 10 times and 100 times together with the ABG for a SSCD with 3.0 mm^2^ opening. The morphology of the three curves in Figure 9 are similar but they differ in magnitude. The ABG for the SSCD falls between the two ABG with OW stiffness increase. This prediction illustrates that it is not possible to distinguish between a SSCD and early stages of otosclerosis based on the ABG alone.

The increase in RW and sometimes also OW stiffness have been used to mitigate the adverse effects of SSCD (19–21). When investigating the predicted effects of increased OW stiffness with SSCD in Figures 8B,D, it can be seen that for AC stimulation, the OW stiffness increase reduces the hearing threshold over a relative wide range of frequencies. For BC stimulation, it has no effect at all. The model predicts that the volume velocity due to fluid inertia, which dominates the response for BC, flows primarily between the RW and the SSCD, and an increase of the stiffness at the OW does not affect this flow. This is illustrated in Figure 8D where the curves for no window stiffness (black dashed line), 10 times OW stiffness (red line) and 100 times OW stiffness (light blue line) nearly overlap. A small increase in RW stiffness (10 times, blue and black lines) reduces the predicted BC response at the lowest frequencies but there is still a 20 dB hyperacusis at around 500 Hz. Once the RW stiffness increase reach 100 times, it reduces the BC sensitivity to near normal values, with a slight depression at the lowest frequencies compared to a healthy ear. These data suggest that for reducing disturbance caused by hyperacusis of internal sounds in SSCD, a reinforcement, or stiffening, at the OW is ineffective, while an increased stiffness at the RW can mitigate the effects once this stiffness increase reach 100 times. Such changes in RW stiffness are predicted to reduce the sensitivity to AC stimulus by more than 20 dB at frequencies <1 kHz. It should be noted that a stiffness increase of the RW by 100 times is significant and would mean that the RW stiffness is 10 times greater than the OW stiffness. Consequently, a stiff plate on the RW would be required to achieve such increased stiffness.

## Conclusions

A lumped element impedance model was able to predict clinical findings in SSCD by both AC and BC stimulation, and gave insight to the mechanisms responsible for the alterations. In general, inertial effects are predicted to be most important for BC hearing in a healthy ear, and the response from fluid inertia becomes even more pronounced in a SSCD case. The SSCD act as a parallel low impedance to the healthy cochlear impedance, which reduces the intra-cochlear sound pressure at low frequencies with a SSCD, leading to worse AC response. The same low impedance from the SSCD improves the volume velocity between the RW and vestibule for BC sound leading to an increased low-frequency BC response. The predicted sound pressure transmission from the cranial cavity to the inner ear via the SSCD seem not to be important for the clinical findings observed.

The normal variability in vestibular aqueduct size do not affect AC hearing and only BC hearing at very low frequencies. The predicted ABG from an early stage of otosclerosis is similar to the ABG from SSCD which indicates the ABGs alone cannot differentiate between these pathologies. The use of window reinforcement to mitigate BC hyperacusis can be effective when the RW is reinforced but has no impact when the OW is reinforced. Such reinforcement do affect AC hearing negatively.

## Data Availability Statement

The raw data supporting the conclusions of this article will be made available by the authors, without undue reservation.

## Author Contributions

SS made the simulations, analyzed the results, and wrote the manuscript.

## Conflict of Interest

The author declares that the research was conducted in the absence of any commercial or financial relationships that could be construed as a potential conflict of interest.
